# Effect of stress‐induced hyperglycemia after non‐traumatic non‐aneurysmal subarachnoid hemorrhage on clinical complications and functional outcomes

**DOI:** 10.1111/cns.13826

**Published:** 2022-03-15

**Authors:** Zeyu Zhang, Yue Zhao, Yibo Liu, Xiaoyu Wang, Houshi Xu, Yuanjian Fang, Anke Zhang, Cameron Lenahan, Yujie Luo, Sheng Chen

**Affiliations:** ^1^ Department of Neurosurgery The Second Affiliated Hospital Zhejiang University School of Medicine Hangzhou China; ^2^ Department of Stomatology The Children's Hospital Zhejiang University School of Medicine National Clinical Research Center for Health Hangzhou China; ^3^ Department of Neurosurgery Shanghai General Hospital Shanghai Jiao Tong University School of Medicine Shanghai China; ^4^ Center for Neuroscience Research Loma Linda University School of Medicine Loma Linda California USA

**Keywords:** complication, functional outcome, hyperglycemia, neuroendocrine, stress, subarachnoid hemorrhage

## Abstract

**Background:**

Despite having an overall benign course, non‐traumatic non‐aneurysmal subarachnoid hemorrhage (naSAH) is still accompanied by a risk of clinical complications and poor outcomes. Risk factors and mechanisms of complications and poor outcomes after naSAH remain unknown. Our aim was to explore the effect of stress‐induced hyperglycemia (SIH) on complication rates and functional outcomes in naSAH patients.

**Methods:**

We retrospectively reviewed patients with naSAH admitted to our institution between 2013 and 2018. SIH was identified according to previous criterion. Symptomatic vasospasm, delayed cerebral infarction, and hydrocephalus were identified as main complications. Outcomes were reviewed using a modified Rankin Scale (mRS) at discharge, 3 months, and 12 months. A statistical analysis was conducted to reveal the associations of SIH with complications and outcomes.

**Results:**

A total of 244 naSAH patients were included in the cohort with 74 (30.3%) SIH. After adjusting for age, gender, hypertension, Hunt and Hess (HH) grade, modified Fisher Scale (mFS), intraventricular hemorrhage (IVH), and subarachnoid blood distribution, SIH was significantly associated with symptomatic vasospasm (*p* < 0.001, 12.176 [4.904–30.231]), delayed cerebral infarction (*p* < 0.001, 12.434 [3.850–40.161]), hydrocephalus (*p* = 0.008, 5.771 [1.570–21.222]), and poor outcome at 12 months (*p* = 0.006, 5.506 [1.632–18.581]), whereas the correlation between SIH and poor outcome at discharge (*p* = 0.064, 2.409 [0.951–6.100]) or 3 months (*p* = 0.110, 2.029 [0.852–4.833]) was not significant. Incorporation of SIH increased the area under curve (AUC) of ROC in the combined model for predicting symptomatic vasospasm (*p* = 0.002), delayed cerebral infarction (*p* = 0.024), hydrocephalus (*p* = 0.037), and 12‐month poor outcome (*p* = 0.087).

**Conclusions:**

SIH is a significant and independent risk factor for symptomatic vasospasm, delayed cerebral infarction, hydrocephalus, and long‐term poor outcome in naSAH patients. Identifying SIH early after naSAH is important for decision‐making and treatment planning.

## Graphical abstract

This is the first study to explore the effect of SIH on complication rates and functional outcomes in naSAH patients. This study found that SIH was a significant and independent risk factor for symptomatic vasospasm, delayed cerebral infarction, hydrocephalus, and long‐term poor outcome in naSAH patients.

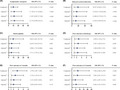

## 1 INTRODUCTION

In approximately 15% of spontaneous subarachnoid hemorrhage (SAH) patients, the source of intracranial hemorrhage could not be determined.^1^, ^2^, ^3^ These are termed non‐traumatic non‐aneurysmal SAH (naSAH).^4^ Compared with aneurysmal SAH (aSAH), naSAH has an overall benign course of disease.^5^ However, some patients with naSAH still develop clinical complications or achieve poor functional outcomes despite their mild condition at admission.^5^, ^6^, ^7^ The risk factors and pathophysiological mechanisms of clinical complications and poor outcomes after naSAH remain unknown.

Stress‐induced hyperglycemia (SIH) is a transient hyperglycemia caused by an acute illness.^8^ It is an adaptive immune–neurohormonal response to stress, and is often associated with increased morbidity and mortality.^8^ Post‐SAH hyperglycemia may cause secondary brain damage and cerebral vasospasm.^9^, ^10^ One previous study showed that post‐aSAH hyperglycemia was associated with the development of symptomatic vasospasm.^11^ Juvela et al. reported that hyperglycemia following aSAH was related to delayed cerebral infarction and hydrocephalus.^12^ Hyperglycemia after aSAH was also found to be related to an increased risk of poor clinical outcome.^12^, ^13^ Additionally, Bian et al. found that a high serum glucose level after aSAH was associated with a high 1‐year mortality.^14^ However, the prognostic value of SIH in patients with naSAH has not yet been established. Moreover, these studies did not differentiate between SIH and established diabetes mellitus (DM).

Therefore, the objective of this study was to examine the effect of SIH on naSAH patients’ complication rates and functional outcomes, and to investigate the prognostic value of SIH for clinical complications and poor outcomes following naSAH.

## 2 METHODS

### 2.1 Patients and management

We retrospectively reviewed patients suffering from naSAH that were admitted to our institution between January 1, 2013 and December 31, 2018. SAH was diagnosed by computed tomography (CT) or lumbar puncture. Non‐traumatic SAH without confirmed bleeding source in cerebral digital subtraction angiography (DSA) examination within 72 h of admission was identified as naSAH.^15^ Additionally, patients who met the following criteria were excluded: (1) history of a head injury; (2) history of DM; (3) missing/lost radiological data; and (4) missing/lost laboratory data. All aspects of this study were approved by the institutional board of the Second Affiliated Hospital of Zhejiang University School of Medicine. With their approval, patient consent was not required in this study.

All patients were treated according to SAH guidelines provided by the Neurocritical Care Society and the American Heart Association.^16^, ^17^ Nimodipine was used to prevent cerebral vasospasm, and intravenous hydration was received to maintain euvolemia. Hemodynamic values were monitored via electrocardiogram at admission. All patients were not treated with insulin during hospitalization. All patients were treated conservatively rather than invasively.

### 2.2 Data collection

The baseline characteristics of the patients were reviewed, including age, gender, body mass index (BMI), as well as history of alcohol, smoking, and hypertension. The Hunt and Hess (HH) grade, modified Fisher Scale (mFS), and development of intraventricular hemorrhage (IVH) were used to assess SAH severity.^18^, ^19^, ^20^, ^21^ Scores ranging from 3 to 5 for HH grade and 3 to 4 for mFS were considered high. According to the subarachnoid blood distribution, the patients were stratified into patients with perimesencephalic subarachnoid hemorrhage (PMH) and patients with non‐PMH (NPMH).^4^, ^5^, ^15^ The characteristics of PMH are as follows: (1) hemorrhage anterior to the midbrain and/or pons; (2) no extension into parenchyma or ventricle deep; (3) incomplete extension into the anterior interhemispheric fissure; (4) possible extension into the basal parts of the sylvian fissures, but not into the lateral sylvian fissures; and (5) no significant IVH. Those who did not meet the bleeding characteristics described above were classified as NPMH. The laboratory data were investigated at admission, including serum glucose, total cholesterol (TC), triglyceride (TG), high‐density lipoprotein cholesterol (HDL‐C), low‐density lipoprotein cholesterol (LDL‐C), sodium, and potassium. If there was more than one glucose measurement at admission, the median would be taken. Serum glucose levels were monitored during hospitalization. SAH‐related complications were reviewed during hospitalization, including symptomatic vasospasm, delayed cerebral infarction, rebleeding, hydrocephalus, and seizure.^22^, ^23^ We followed up with all patients in an outpatient clinic or by phone calls. The modified Rankin Scale (mRS) of patients at discharge, 3 months, and 12 months were investigated to assess the functional outcomes.^24^

According to the latest consensus from the American Association of Clinical Endocrinologists and American Diabetes Association, SIH was defined as at least one of the following criteria: (1) an admission serum glucose level of 7.8 mmol/L (140 mg/dl) or more; (2) an in‐hospital fasting serum glucose level of 7.0 mmol/L (126 mg/dl) or more on 2 or more determinations; and (3) a random serum glucose level of 11.1 mmol/L (200 mg/dl) or more without a prior history of DM.^25^

### 2.3 Outcome measurements

The primary outcomes included the development of clinical complications and poor outcomes. The main clinical complications included symptomatic vasospasm, delayed cerebral infarction, and hydrocephalus. Symptomatic vasospasm referred to either a focal neurological impairment or a decrease of at least 2 points on the Glasgow Coma Scale (GCS) lasting for at least 1 h, which was not immediately apparent after SAH onset, and was not attributable to other causes.^26^ Delayed cerebral infarction was diagnosed as a new infarction emerging on CT or magnetic resonance imaging (MRI), which had not originally been present within the first 24–48 h after SAH onset, and was not attributable to other causes.^26^ Hydrocephalus was defined as an expansion of the ventricular system on neuroimaging without obstructive cause or typical clinical presentation.^27^ Functional outcomes were reviewed at discharge, 3 months, and 12 months using an mRS. Due to the overall favorable prognosis of naSAH patients, mRS scores ranging from 2 to 6 were considered poor outcome.^15^ Two senior neurologists independently evaluated all clinical complications and functional outcomes of the patients. If there was a divergence, a third examiner would be used.

### 2.4 Statistical analysis

Statistical analysis was performed using IBM‐SPSS V24.0 (SPSS Inc) with the statistical significance set at *p* < 0.05. The Shapiro–Wilk test was performed to assess data distribution. Normally distributed variables were expressed as means ± standard deviations (SD). Categorical variables were expressed as the number of patients (percentage). Student's *t*‐test was used to compare the normally distributed variables. The χ^2^‐test or Fisher's exact test was used to compare the categorical variables. Variables with a *p* value <0.10 in univariate analysis were included in the multivariate logistic regression model to identify the independent risk factors of clinical complications and poor outcomes. Combined models were established to assess the predictive value of SIH for complications and poor outcomes. Odds ratio (OR) and 95% confidence interval (Cl) were calculated. Receiver operating curve (ROC) was drawn using Prism 8 (GraphPad Software, Inc). The area under curve (AUC) was calculated to assess the ability of the models to predict clinical complications and poor outcomes. The AUC of the models was compared using MedCalc v20.0.22.

## 3 RESULTS

### 3.1 Patient characteristics

There were 296 patients diagnosed with naSAH in this study. Thirteen patients had a history of head injury. Twenty‐two patients suffered from DM. Ten patients were missing radiological data and seven patients were missing laboratory data. Thus, 244 patients were included in the final cohort, with 74 (30.3%) suffering from SIH (Figure S1). Among the patients, 108 (44.3%) were women, and the average age was 55.7 ± 11.2 years.

Table 1 shows the baseline characteristics, complications, and outcomes of the patients. Patients with SIH had a higher age (*p* = 0.004) and a higher proportion of hypertension (*p* = 0.002) and NPMH (*p* = 0.010). Regarding SAH severity, the HH grade 3–5 (*p* < 0.001), mFS 3–4 (*p* = 0.003), and IVH (*p* = 0.026) all correlated with SIH. In addition, SIH patients were more likely to develop SAH‐related complications, including symptomatic vasospasm, delayed cerebral infarction, and hydrocephalus (all *p* < 0.001). They also had a higher proportion of mRS 2–6 at discharge (*p* = 0.013), 3 months (*p* < 0.001) and 12 months (*p* < 0.001). Figure S2 shows the subarachnoid blood distribution characteristic, HH grade, mFS, and IVH incidence of SIH and non‐SIH patients. The main in‐hospital complication rates and mRS distribution of the two groups of patients are shown in Figure S3. The characteristics of PMH and NPMH patients are shown in Table S1.

**TABLE 1:** Baseline characteristics, complications, and outcomes of SIH and non‐SIH patients

Variable	Total (*n *= 244)		*p* value
	SIH (*n *= 74)	non‐SIH (*n *= 170)	
Age, year	58.8 ± 10.5	54.4 ± 11.2	0.004
Gender, female	39 (52.7)	69 (40.6)	0.080
Alcohol	27 (36.5)	67 (39.4)	0.666
Smoke	25 (33.8)	63 (37.1)	0.624
Hypertension	35 (47.3)	46 (27.1)	0.002
NPMH	33 (44.6)	47 (27.6)	0.010
HH grade 3–5	19 (25.7)	11 (6.5)	<0.001
mFS 3–4	26 (35.1)	30 (17.6)	0.003
IVH	23 (31.1)	31 (18.2)	0.026
BMI, kg/m^2^	23.9 ± 3.1	23.6 ± 2.7	0.474
Glucose, mmol/L	9.24 ± 2.03	6.35 ± 0.78	<0.001
TC, mmol/L	4.86 ± 1.05	4.80 ± 1.05	0.732
TG, mmol/L	1.51 ± 0.88	1.39 ± 0.72	0.383
HDL‐C, mmol/L	1.34 ± 0.34	1.28 ± 0.29	0.221
LDL‐C, mmol/L	2.59 ± 0.81	2.68 ± 0.81	0.454
Sodium, mmol/L	139.3 ± 4.0	139.0 ± 3.3	0.491
Potassium, mmol/L	3.73 ± 0.47	3.81 ± 0.36	0.206
Symptomatic vasospasm	41 (55.4)	19 (11.2)	<0.001
Delayed cerebral infarction	27 (36.5)	5 (2.9)	<0.001
Rebleeding	3 (4.1)	3 (1.8)	0.541
Hydrocephalus	14 (18.9)	4 (2.4)	<0.001
Seizure	0 (0)	3 (1.8)	0.555
Poor outcome at discharge	67 (90.5)	131 (77.1)	0.013
Poor outcome at 3 months	19 (25.7)	15 (8.8)	<0.001
Poor outcome at 12 months	14 (18.9)	5 (2.9)	<0.001

Abbreviations: BMI, body mass index; HDL‐C, high‐density lipoprotein cholesterol; HH, Hunt and Hess; IVH, intraventricular hemorrhage; LDL‐C, low‐density lipoprotein cholesterol; mFS, modified Fisher scale; NPMH, non‐perimesencephalic subarachnoid hemorrhage; SIH, stress‐induced hyperglycemia; TC, total cholesterol; TG, triglyceride.

### 3.2 Association of variables with clinical complications and functional outcomes

The associations between variables and clinical complications are shown in Table 2. The development of symptomatic vasospasm was significantly associated with NPMH, HH grade 3–5, mFS 3–4, IVH, higher admission serum glucose levels, and SIH (all *p* < 0.001). Patients with delayed cerebral infarction had higher age (*p* = 0.028) and admission serum glucose levels (*p* < 0.001), lower serum potassium levels (*p* = 0.007), and a higher proportion of hypertension, NPMH, HH grade 3–5, mFS 3–4, IVH, and SIH (all *p* < 0.001). The hydrocephalus group was significantly related to higher serum glucose levels at admission (*p* = 0.001) and a higher proportion of hypertension (*p* = 0.002), NPMH (*p* < 0.001), HH grade 3–5 (*p* < 0.001), mFS 3–4 (*p* < 0.001), IVH (*p* = 0.003), and SIH (*p* < 0.001).

**TABLE 2:** Association of variables with symptomatic vasospasm, delayed cerebral infarction, and hydrocephalus

Variable	Symptomatic vasospasm		*p* value	Delayed cerebral infarction		*p* value	Hydrocephalus		*p* value
	Yes (*n* = 60)	No (*n* = 184)		Yes (*n* = 32)	No (*n* = 212)		Yes (*n* = 18)	No (*n* = 226)	
Age, year	58.0 ± 10.6	55.0 ± 11.3	0.068	59.8 ± 10.9	55.1 ± 11.1	0.028	59.1 ± 8.9	55.5 ± 11.3	0.191
Gender, female	26 (43.3)	82 (44.6)	0.868	16 (50.0)	92 (43.4)	0.483	7 (38.9)	101 (44.7)	0.633
Alcohol	19 (31.7)	75 (40.8)	0.209	11 (34.4)	83 (39.2)	0.605	6 (33.3)	88 (38.9)	0.638
Smoke	18 (30.0)	70 (38.0)	0.260	12 (37.5)	76 (35.8)	0.856	5 (27.8)	83 (36.7)	0.447
Hypertension	26 (43.3)	55 (29.9)	0.055	20 (62.5)	61 (28.8)	<0.001	12 (66.7)	69 (30.5)	0.002
NPMH	42 (70.0)	38 (20.7)	<0.001	24 (75.0)	56 (26.4)	<0.001	15 (83.3)	65 (28.8)	<0.001
HH grade 3–5	22 (36.7)	8 (4.3)	<0.001	17 (53.1)	13 (6.1)	<0.001	9 (50.0)	21 (9.3)	<0.001
mFS 3–4	36 (60.0)	20 (10.9)	<0.001	19 (59.4)	37 (17.5)	<0.001	13 (72.2)	43 (19)	<0.001
IVH	27 (45.0)	27 (14.7)	<0.001	17 (53.1)	37 (17.5)	<0.001	9 (50.0)	45 (19.9)	0.003
BMI, kg/m^2^	23.9 ± 2.9	23.7 ± 2.8	0.613	23.4 ± 3.1	23.8 ± 2.8	0.415	23.0 ± 2.8	23.8 ± 2.8	0.232
Glucose, mmol/L	8.82 ± 2.42	6.70 ± 1.26	<0.001	9.63 ± 2.72	6.86 ± 1.37	<0.001	9.23 ± 2.35	7.06 ± 1.72	0.001
SIH	41 (68.3)	33 (17.9)	<0.001	27 (84.4)	47 (22.2)	<0.001	14 (77.8)	60 (26.5)	<0.001
TC, mmol/L	4.76 ± 1.01	4.84 ± 1.06	0.667	4.87 ± 0.93	4.81 ± 1.06	0.793	5.02 ± 1.05	4.80 ± 1.05	0.434
TG, mmol/L	1.47 ± 0.73	1.41 ± 0.79	0.640	1.56 ± 0.74	1.41 ± 0.78	0.390	1.33 ± 0.49	1.44 ± 0.79	0.603
HDL‐C, mmol/L	1.33 ± 0.35	1.28 ± 0.29	0.289	1.27 ± 0.39	1.30 ± 0.30	0.724	1.31 ± 0.29	1.29 ± 0.31	0.856
LDL‐C, mmol/L	2.58 ± 0.82	2.68 ± 0.81	0.443	2.71 ± 0.82	2.65 ± 0.81	0.717	2.80 ± 0.79	2.64 ± 0.81	0.466
Sodium, mmol/L	139.4 ± 4.2	138.9 ± 3.3	0.358	139.1 ± 5.1	139.1 ± 3.3	0.995	138.5 ± 5.4	139.1 ± 3.4	0.479
Potassium, mmol/L	3.71 ± 0.44	3.81 ± 0.38	0.109	3.61 ± 0.50	3.81 ± 0.38	0.007	3.68 ± 0.48	3.79 ± 0.39	0.251

Abbreviations: BMI, body mass index; HDL‐C, high‐density lipoprotein cholesterol; HH, Hunt and Hess; IVH, intraventricular hemorrhage; LDL‐C, low‐density lipoprotein cholesterol; mFS, modified Fisher scale; NPMH, non‐perimesencephalic subarachnoid hemorrhage; SIH, stress‐induced hyperglycemia; TC, total cholesterol; TG, triglyceride.

Table 3 shows the associations between variables and functional outcomes. There were significant correlations between poor outcome at discharge and higher admission serum glucose levels (*p* = 0.048), SIH (*p* = 0.013), NPMH (*p* = 0.002), and higher SAH severity, including HH grade 3–5 (*p* = 0.038), mFS 3–4 (*p* = 0.002), and IVH (*p* = 0.002). Similar results were observed in 3‐month and 12‐month outcomes (all *p* < 0.05).

**TABLE 3:** Association of variables with functional outcomes at discharge, 3 months, and 12 months

Variable	Poor outcome at discharge		*p* value	Poor outcome at 3 months		*p* value	Poor outcome at 12 months		*p* value
	Yes (*n* = 198)	No (*n* = 46)		Yes (*n* = 34)	No (*n* = 210)		Yes (*n* = 19)	No (*n* = 225)	
Age, year	56.2 ± 10.8	53.8 ± 12.5	0.188	57.6 ± 11.6	55.4 ± 11.1	0.283	59.1 ± 11.1	55.5 ± 11.1	0.171
Gender, female	85 (42.9)	23 (50.0)	0.384	14 (41.2)	94 (44.8)	0.696	4 (21.1)	104 (46.2)	0.060
Alcohol	77 (38.9)	17 (37.0)	0.808	10 (29.4)	84 (40.0)	0.239	7 (36.8)	87 (38.7)	0.875
Smoke	72 (36.4)	16 (34.8)	0.841	13 (38.2)	75 (35.7)	0.776	9 (47.4)	79 (35.1)	0.285
Hypertension	65 (32.8)	16 (34.8)	0.800	16 (47.1)	65 (31.0)	0.064	10 (52.6)	71 (31.6)	0.061
NPMH	74 (37.4)	6 (13.0)	0.002	22 (64.7)	58 (27.6)	<0.001	15 (78.9)	65 (28.9)	<0.001
HH grade 3–5	29 (14.6)	1 (2.2)	0.038	15 (44.1)	15 (7.1)	<0.001	10 (52.6)	20 (8.9)	<0.001
mFS 3–4	54 (27.3)	2 (4.3)	0.002	17 (50.0)	39 (18.6)	<0.001	12 (63.2)	44 (19.6)	<0.001
IVH	52 (26.3)	2 (4.3)	0.002	13 (38.2)	41 (19.5)	0.015	9 (47.4)	45 (20.0)	0.006
BMI, kg/m^2^	23.8 ± 2.8	23.4 ± 2.6	0.380	24.3 ± 3.2	23.6 ± 2.7	0.230	23.9 ± 3.6	23.7 ± 2.7	0.843
Glucose, mmol/L	7.34 ± 1.94	6.74 ± 1.38	0.048	8.78 ± 3.14	6.97 ± 1.41	0.002	9.56 ± 2.89	7.03 ± 1.60	0.001
SIH	67 (33.8)	7 (15.2)	0.013	19 (55.9)	55 (26.2)	<0.001	14 (73.7)	60 (26.7)	<0.001
TC, mmol/L	4.82 ± 1.07	4.81 ± 0.93	0.942	4.75 ± 0.83	4.83 ± 1.08	0.721	4.79 ± 0.91	4.82 ± 1.06	0.904
TG, mmol/L	1.42 ± 0.72	1.47 ± 0.96	0.684	1.44 ± 0.70	1.43 ± 0.78	0.926	1.41 ± 0.69	1.43 ± 0.78	0.919
HDL‐C, mmol/L	1.31 ± 0.30	1.23 ± 0.32	0.159	1.36 ± 0.36	1.28 ± 0.30	0.242	1.38 ± 0.38	1.29 ± 0.30	0.212
LDL‐C, mmol/L	2.65 ± 0.85	2.67 ± 0.63	0.865	2.60 ± 0.75	2.67 ± 0.82	0.691	2.60 ± 0.86	2.66 ± 0.81	0.753
Sodium, mmol/L	139.1 ± 3.7	138.9 ± 3.1	0.752	139.0 ± 4.8	139.1 ± 3.3	0.885	138.7 ± 5.3	139.1 ± 3.4	0.674
Potassium, mmol/L	3.79 ± 0.39	3.74 ± 0.46	0.419	3.68 ± 0.50	3.80 ± 0.38	0.195	3.71 ± 0.52	3.79 ± 0.39	0.410

Abbreviations: BMI, body mass index; HDL‐C, high‐density lipoprotein cholesterol; HH, Hunt and Hess; IVH, intraventricular hemorrhage; LDL‐C, low‐density lipoprotein cholesterol; mFS, modified Fisher scale; NPMH, non‐perimesencephalic subarachnoid hemorrhage; SIH, stress‐induced hyperglycemia; TC, total cholesterol; TG, triglyceride.

The results of univariate logistic regression analysis for predicting clinical complications and functional outcomes are shown in Table S2 and Table S3.

### 3.3 Effect of SIH on clinical complications and functional outcomes

**FIGURE 1:**
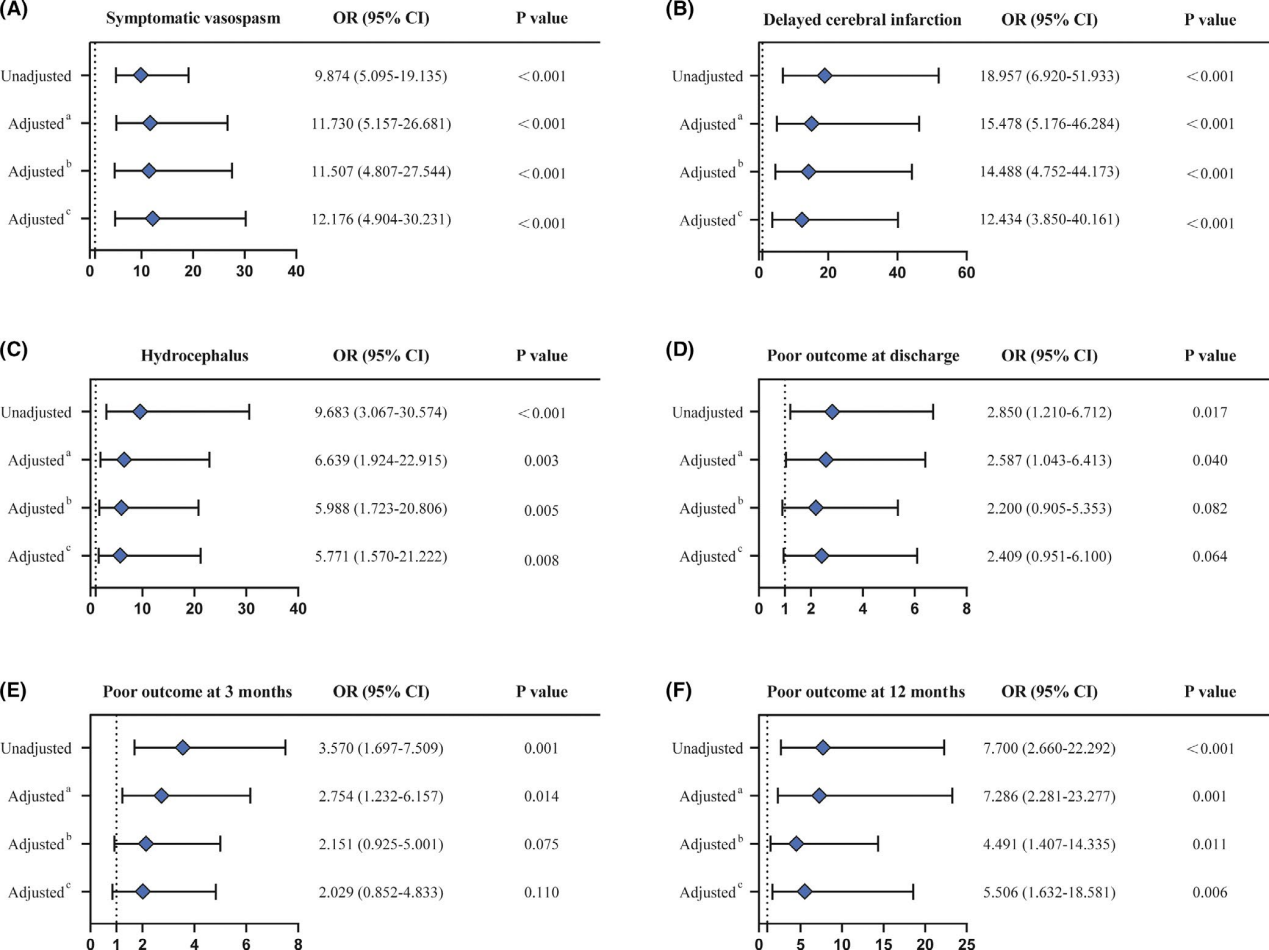
Unadjusted and adjusted OR for SIH to evaluate the effect of SIH on clinical complications and functional outcomes. After adjusting for age, gender, hypertension, HH grade, mFS, IVH, and subarachnoid blood distribution characteristic, SIH was still significantly associated with symptomatic vasospasm (A, *p* < 0.001), delayed cerebral infarction (B, *p* < 0.001), hydrocephalus (C, *p* = 0.008), and 12‐month poor outcome (F, *p* = 0.006), but was not significantly associated with discharge poor outcome (D, *p* = 0.064) and 3‐month poor outcome (E, *p* = 0.110). ^a^Adjusted for age, gender, hypertension, and subarachnoid blood distribution characteristic. ^b^Adjusted for HH grade, mFS, IVH, and subarachnoid blood distribution characteristic. ^c^Adjusted for age, gender, hypertension, HH grade, mFS, IVH, and subarachnoid blood distribution characteristic. HH, Hunt and Hess; IVH, intraventricular hemorrhage; mFS, modified Fisher scale; OR, odds ratio; SIH, stress‐induced hyperglycemia

SIH was found to be significantly associated with clinical complications and adverse outcomes in our cohort. Therefore, we evaluated the effect of SIH on clinical complications and functional outcomes in multivariate logistic regression analysis. As is depicted in Figure 1, when accompanied by SIH, there was a 12.176 (*p* < 0.001, 95% CI 4.904–30.231) increase in the odds of developing symptomatic vasospasm, a 12.434 (*p* < 0.001, 95% CI 3.850–40.161) increase in the odds of developing delayed cerebral infarction, and a 5.771 (*p* = 0.008, 95% CI 1.570–21.222) increase in the odds of developing hydrocephalus after adjustment for covariates, including age, gender, hypertension, HH grade, mFS, IVH, and subarachnoid blood distribution characteristic. Regarding functional outcomes, there were no significant associations between SIH and poor outcome at discharge (*p* = 0.064, OR 2.409, 95% CI 0.951–6.100) or at 3 months (*p* = 0.110, OR 2.029, 95% CI 0.852–4.833) after adjusting for the above covariates. Interestingly, SIH was significantly and independently associated with poor outcome at 12 months (*p* = 0.006, OR 5.506, 95% CI 1.632–18.581).

### 3.4 Prognostic value of SIH for clinical complications and functional outcomes

The combined models were constructed to predict clinical complications and functional outcomes. The results are displayed in Table 4. Model 1 was obtained by incorporating variables with a *p* value <0.10 in univariate analysis and using a forward stepwise method of multivariate logistic regression analysis. SIH was incorporated in the prediction of symptomatic vasospasm (*p* < 0.001, OR 11.507, 95% CI 4.807–27.544), delayed cerebral infarction (*p* < 0.001, OR 13.874, 95% CI 4.424–43.507), hydrocephalus (*p* = 0.003, OR 6.474, 95% CI 1.915–21.894), discharge poor outcome (*p* = 0.038, OR 2.512, 95% CI 1.051–6.001), 3‐month poor outcome (*p* = 0.006, OR 2.980, 95% CI 1.376–6.452), and 12‐month poor outcome (*p* = 0.010, OR 4.556, 95% CI 1.447–14.345). Model 2 was obtained in the same manner, but with exclusion of the variable SIH.

**TABLE 4:** Multivariate logistic regression models for predicting clinical complications and functional outcomes

Variable	Model 1 (SIH included)		Model 2 (SIH excluded)	
	OR (95% CI)	*p* value	OR (95% CI)	*p* value
Symptomatic vasospasm				
SIH	11.507 (4.807–27.544)	<0.001	N/A	N/A
NPMH	3.096 (1.079–8.884)	0.036	2.724 (1.086–6.830)	0.033
HH grade 3–5	3.417 (1.002–11.658)	0.050	4.908 (1.744–13.812)	0.003
mFS 3–4	5.007 (1.661–15.091)	0.004	4.047 (1.550–10.566)	0.004
IVH	2.636 (1.045–6.650)	0.040	2.510 (1.119–5.629)	0.026
Delayed cerebral infarction				
SIH	13.874 (4.424–43.507)	<0.001	N/A	N/A
Hypertension	3.200 (1.149–8.912)	0.026	4.109 (1.632–10.346)	0.003
NPMH	5.447 (1.878–15.796)	0.002	5.330 (2.035–13.963)	0.001
HH grade 3–5	7.457 (2.373–23.430)	0.001	9.760 (3.578–26.624)	N/A
Hydrocephalus				
SIH	6.474 (1.915–21.894)	0.003	N/A	N/A
Hypertension	3.822 (1.221–11.970)	0.021	4.491 (1.522–13.252)	0.007
mFS 3–4	9.389 (2.944–29.940)	<0.001	10.967 (3.612–33.292)	<0.001
Poor outcome at discharge				
SIH	2.512 (1.051–6.001)	0.038	N/A	N/A
mFS 3–4	N/A	N/A	6.436 (1.487–27.852)	0.013
IVH	7.092 (1.650–30.478)	0.008	6.024 (1.389–26.127)	0.016
Poor outcome at 3 months				
SIH	2.980 (1.376–6.452)	0.006	N/A	N/A
NPMH	4.195 (1.918–9.173)	<0.001	2.990 (1.296–6.899)	0.010
HH grade 3–5	N/A	N/A	6.806 (2.739–16.911)	<0.001
Poor outcome at 12 months				
SIH	4.556 (1.447–14.345)	0.010	N/A	N/A
NPMH	5.086 (1.483–17.445)	0.010	5.580 (1.665–18.706)	0.005
HH grade 3–5	4.144 (1.322–12.988)	0.015	6.234 (2.116–18.366)	0.001

Note

Model 1 was obtained by incorporating variables with a *p* value <0.10 in univariate analysis and using a forward stepwise method of multivariate logistic regression analysis. Model 2 was obtained in the same manner, but excluding the variable SIH.

Abbreviations: Cl, confidence interval; HH, Hunt and Hess; IVH, intraventricular hemorrhage; mFS, modified Fisher scale; N/A, not applicable; NPMH, non‐perimesencephalic subarachnoid hemorrhage; OR, odds ratio; SIH, stress‐induced hyperglycemia.

**FIGURE 2:**
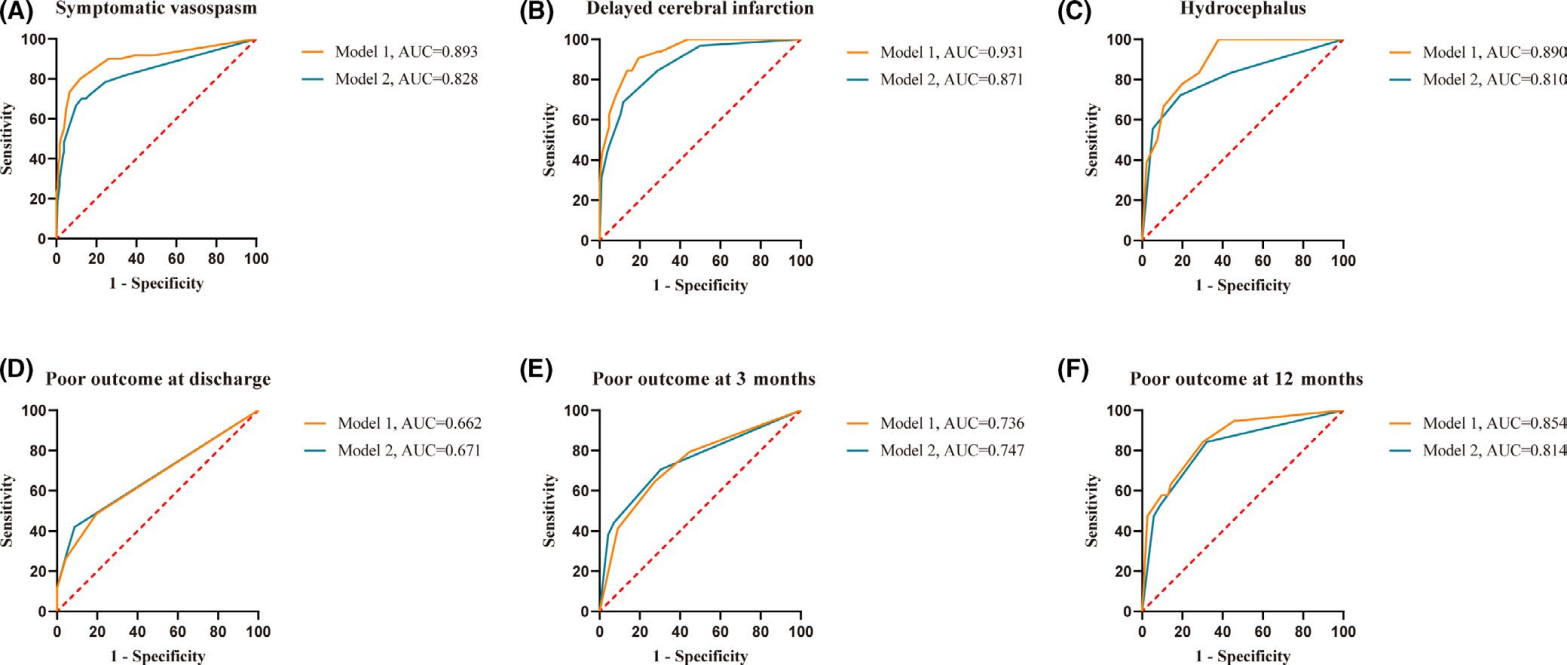
ROC curve for combined model to predict clinical complications and functional outcomes. In predicting symptomatic vasospasm (A, *p* = 0.002), delayed cerebral infarction (B, *p* = 0.024), and hydrocephalus (C, *p* = 0.037), model 1 had a significantly higher AUC than model 2. In predicting discharge poor outcome (D, *p* = 0.775) and 3‐month poor outcome (E, *p* = 0.659), the AUC of model 1 was not higher than that of model 2. In the prediction of 12‐month poor outcome (F, *p* = 0.087), the AUC of model 1 was higher than that of model 2, although it was not statistically significant. AUC, area under curve; ROC, receiver operating curve

Hosmer–Lemeshow test was performed to assess the goodness of fit of the models (all *p* > 0.05). ROC analysis was performed to evaluate the predictive ability of the models (Figure 2). In the ROC analysis, model 1 had a significantly higher AUC compared with model 2 for the prediction of symptomatic vasospasm (0.893 [0.848–0.929] vs. 0.828 [0.774–0.873]; *p* = 0.002). Similar results were observed when predicting delayed cerebral infarction (0.931 [0.892–0.959] vs. 0.871 [0.823–0.911]; *p* = 0.024) and hydrocephalus (0.890 [0.843–0.926] vs. 0.810 [0.755–0.857]; *p* = 0.037). However, in the prediction of poor outcomes at discharge (0.662 [0.599–0.721] vs. 0.671 [0.609–0.730]; *p* = 0.775) and 3 months (0.736 [0.676–0.790] vs. 0.747 [0.687–0.800]; *p* = 0.659), the AUC of model 1 was not higher than that of model 2. It is worth noting that, although not statistically significant, the AUC of model 1 was higher than that of model 2 in predicting 12‐month poor outcome (0.854 [0.804–0.896] vs. 0.814 [0.759–0.861]; *p* = 0.087). Thus, incorporation of SIH increased the ability of the model for the prediction of symptomatic vasospasm, delayed cerebral infarction, hydrocephalus, and 12‐month poor outcome.

## 4 DISCUSSION

Although previous studies have explored risk factors for adverse outcomes in naSAH patients, including high clinical and radiological severity, cerebral edema, and NPMH, the relationship between SIH and outcomes has not been investigated.^28^, ^29^ To our knowledge, this is the first study to explore the association of SIH with complication rates and functional outcomes in naSAH. This study found that SIH after naSAH was significantly and independently associated with the development of symptomatic vasospasm, delayed cerebral infarction, hydrocephalus, and a poor outcome at 12 months after adjusting for demographic data, hypertension history, subarachnoid blood distribution characteristic, and SAH severity. Taking SIH into consideration with risk factors improved the prediction of symptomatic vasospasm, delayed cerebral infarction, hydrocephalus, and 12‐month poor outcome after naSAH, although it had limited benefits in the prediction of poor outcomes at discharge or at 3 months. These findings highlight the importance of considering SIH in the decision‐making algorithm and treatment planning following naSAH.

SIH is a transient hyperglycemia after acute illness or injury caused by the activation of stress‐neuroendocrine axis.^8^ Previous research suggested that post‐stroke SIH may be a biomarker of stroke severity.^30^, ^31^ One meta‐analysis involving 16 studies showed that 69% (range, 29 to 100%) of patients suffered a SIH after aSAH.^13^ The proportion of SIH in our study cohort (30.3%) was much lower than this proportion, reflecting the low severity of naSAH. In one study, SIH occurred in 32.0% of naSAH patients, which was similar to the proportion in our cohort.^32^ An early study confirmed a significant correlation between high serum glucose levels and high clinical severity assessed by HH grade in aSAH patients (*p* = 0.001).^31^ In addition, Santucci et al. reported that SIH after aSAH was significantly associated with radiologically estimated intracranial blood volume (*p* < 0.001).^33^ Our results in naSAH cohort were consistent with these findings. In our study, patients with SIH had higher HH grade (*p* = 0.001), mFS (*p* = 0.003) and a higher proportion of IVH (*p* = 0.026; Figure S2). Additionally, in the characteristic of subarachnoid blood distribution, the proportion of NPMH in SIH patients was higher than that of non‐SIH patients (*p* = 0.010), which may be due to the fact that NPMH was more similar to aSAH with a higher severity.^4^, ^5^ These results may indicate the systemic stress response caused by severe brain injury after SAH.

Several studies have explored the relationship between hyperglycemia and complication rates and adverse outcomes after aSAH. Badjatia et al. found that mean serum glucose levels during hospitalization correlated with the development of symptomatic vasospasm after aSAH (*p* < 0.001).^11^ Juvela and colleagues reported that hyperglycemia following aSAH were related to delayed cerebral infarction and hydrocephalus.^12^ A meta‐analysis incorporated eight studies for the analysis of the association between hyperglycemia and clinical outcome after aSAH and found that post‐aSAH hyperglycemia was associated with an increased risk of poor clinical outcome at 3 or 6 months.^13^ These results supported our findings. However, these studies only identified patients with hyperglycemia, but did not differentiate between SIH patients and DM patients. Since pre‐existing hyperglycemia before SAH onset could not reflect the activation of stress response caused by SAH, our study excluded patients with a history of DM. Moreover, these studies only described the association of hyperglycemia after SAH with functional outcomes within 6 months, but did not explore its relationship with long‐term outcomes. Our study identified patients with SIH based on the latest consensus from the American Association of Clinical Endocrinologists and American Diabetes Association, and demonstrated the independent association of SIH with symptomatic vasospasm (*p* < 0.001), delayed cerebral infarction (*p* < 0.001), and hydrocephalus (*p* = 0.008) in the naSAH cohort. After adjusting for demographic data, hypertension history, subarachnoid blood distribution characteristic, and SAH severity, SIH was significantly associated with adverse outcomes at 12 months (*p* = 0.006), although no significant correlation was found between SIH and poor outcomes at discharge (*p* = 0.064) or at 3 months (*p* = 0.110; Figure 1). The poor role of SIH in predicting short‐term prognosis may be due to the high proportion of patients with short‐term poor outcomes. In our cohort, 81.1% (198/244) of patients had a poor outcome at discharge. However, most (90.4%) of them had an improved outcome at 12 months. The significant association between SIH and long‐term poor prognosis may implicate the chronic impairment of SIH in naSAH patients and suggest its value in predicting truly poor outcomes. On the other hand, the association between SIH and short‐term poor prognosis may become significant in larger cohorts.

Traditionally, prediction of poor outcomes after aSAH primarily depends on aneurysm size and location and SAH severity assessed by HH grade and mFS.^34^, ^35^ However, aneurysm size and location are not applicable in the prediction of poor outcomes in naSAH. Moreover, naSAH patients usually have minor SAH severity at admission. In our cohort of naSAH, patients with HH grade 1–2 and mFS 0–2 accounted for 87.7% and 77.0%, respectively. Therefore, it is relatively difficult to predict poor outcomes in naSAH patients compared to aSAH patients. Although SIH may be useful in predicting poor outcomes in both aSAH and naSAH patients,^13^ the benefits for naSAH patients are obviously greater.

The deleterious effects of activation of stress‐neuroendocrine axis after SAH may explain the correlation between SIH and adverse outcomes. The stress response can induce the activation of hypothalamus–pituitary–adrenal axis and sympathetic autonomic nervous system, as well as induce the secretion of glucagon, catecholamines, and corticosteroids.^8^ The metabolic disorders caused by these factors may lead to inflammation, systemic damage, and various complications.^8^ Sympathetic activation and serum catecholamine elevation after aSAH has been confirmed in previous studies, and were found to be related to symptomatic vasospasm and unfavorable outcomes.^36^, ^37^ It was found that inhibition of sympathetic activity by beta‐blockers could reduce cerebral vasospasm rates and improve functional outcomes after aSAH.^38^, ^39^ In an animal study, inhibition of central sympathetic nerve activation through renal denervation significantly prevented cerebral vasospasm after SAH.^40^ In addition, hyperglycemia may aggravate early brain injury (EBI) after SAH. Currently, new insight suggests that EBI within 72 h after SAH onset may lay the foundation for subsequent pathophysiological changes and poor outcomes of patients. The pathological mechanisms of EBI include oxidative stress, platelet activation, inflammation, and neuronal apoptosis.^41^, ^42^, ^43^, ^44^, ^45^, ^46^ Additionally, new studies showed that microthrombi formation, vascular injury and iron homeostasis disorder were also important mechanisms of EBI after SAH.^47^, ^48^ One animal experiment found that hyperglycemia could increase reactive oxygen species (ROS) production through activating protein kinase C after stroke, thereby exacerbating oxidative stress.^49^ In another study, hyperglycemia aggravated neuronal apoptosis through the activation of extrinsic caspase cascade via extracellular regulated kinase (ERK) signal pathway after experimental SAH.^9^ In addition, hyperglycemia was also found to be related to the activation of platelets and the increase of pro‐inflammatory cytokines.^50^ Moreover, a recent animal study suggested that hyperglycemia could exacerbate cerebral hemorrhagic transformation by interfering with gut microbiota.^51^

In the present study, SIH was a significant and independent risk factor of symptomatic vasospasm, delayed cerebral infarction, hydrocephalus, and 12‐month poor outcome in patients with naSAH. Taking SIH into consideration with risk factors improved the prediction of symptomatic vasospasm, delayed cerebral infarction, hydrocephalus, and 12‐month poor outcome. Biomarkers for predicting complications and poor outcomes after naSAH have not yet been established. Currently, prediction of unfavorable outcomes after naSAH primarily still depends on the severity of SAH, which is assessed using HH grade, mFS, and occurrence of IVH. However, naSAH patients often have minor SAH severity at admission.^5^, ^6^, ^7^ Thus, SAH severity has limited value in predicting poor outcomes in this population. Since the identification of SIH could easily be performed in any institution, it should be an important reference for predicting complications and poor outcomes of naSAH patients. In addition, this study suggested that the stress response and resulting hyperglycemia after naSAH may be harmful to patients. Some previous data also supported the possibility that maintaining normoglycemia may be a strategy to improve outcomes after SAH.^52^, ^53^ Therefore, we recommend proper glycemic management for naSAH patients with SIH.

This study had several limitations. First, we did not detect the patients’ serum endocrine hormones, such as cortisol and catecholamines, to better explore the activation of the stress‐neuroendocrine axis after naSAH. Second, we lost the data regarding glycated hemoglobin (HbA1c) of the patients to define SIH more precisely through relative hyperglycemia. To address this problem, we defined SIH through the serum glucose levels at admission and during hospitalization according to the latest consensus from the American Association of Clinical Endocrinologists and American Diabetes Association. The proportion of naSAH patients in our cohort who developed SIH (30.3%) was comparable to a previous study that defined SIH by relative hyperglycemia (32.0%).^32^ Third, the assessments of SAH severity and outcomes may be subjective. To solve this problem, two senior neurologists performed the evaluations independently. If there was a divergence, a third examiner would be used. Fourth, some patients may have more than one glucose measurement at admission, which may bias the identification of SIH. To address this problem, we would take the median if there was more than one glucose measurement at admission. Fifth, our study did not reveal whether interventions on hyperglycemia after naSAH could assist in reducing risk of the development of complications and poor outcomes. Finally, our study was a single‐center retrospective study. Further multi‐center prospective studies are needed to verify our findings.

## 5 CONCLUSIONS

This study found that SIH was a significant and independent risk factor for symptomatic vasospasm, delayed cerebral infarction, hydrocephalus, and long‐term poor outcomes in patients with naSAH. SIH was useful for predicting complications and long‐term prognosis of naSAH, although its benefit in the prediction of short‐term prognosis was limited. In addition, this study may allude to the underlying mechanism of stress‐neuroendocrine axis in the pathogenesis of naSAH. Our findings highlight the importance of identifying SIH early after naSAH for decision‐making and treatment planning.

## CONFLICT OF INTEREST

The authors declare that they have no conflicts of interest.

## AUTHOR CONTRIBUTIONS

ZYZ wrote the manuscript; YZ and SC designed the study; AKZ and XYW collected the study data; YJF, SC, and CL revised the manuscript; YBL, HSX, and YJL participated in the design and coordination of the study. All authors read and approved the final version of the manuscript.

## Supporting information

Figure S1

Figure S2

Figure S3

Table S1

Table S2

Table S3

## Data Availability

The data that support the findings of this study are available from the corresponding author upon reasonable request.
