# Supplementary material for: Effect of stress‐induced hyperglycemia after non‐traumatic non‐aneurysmal subarachnoid hemorrhage on clinical complications and functional outcomes
Source: CNS Neurosci Ther. 2022 Mar 15;28(6):942–52. doi: 10.1111/cns.13826 (PMC9062555; doi:10.1111/cns.13826)
Supplement: Supplementary file 4 — Table S1 [file CNS-28-942-s001.docx]

|  |  |  | **Total (n=244)** | |  |  |  |
| --- | --- | --- | --- | --- | --- | --- | --- |
| **Variable** | | | **PMH (n=164)** | **NPMH (n=80)** | **P value** | | |
| Age, yr | | | 55.3±10.8 | 56.7±11.9 | 0.342 | | |
| Gender, female | | | 78 (47.6) | 30 (37.5) | 0.137 | | |
| Alcohol | | | 63 (38.4) | 31 (38.8) | 0.960 | | |
| Smoke | | | 59 (36.0) | 29 (36.3) | 0.967 | | |
| Hypertension | | | 52 (31.7) | 29 (36.3) | 0.479 | | |
| HH grade 3-5 | | | 8 (4.9) | 22 (27.5) | < 0.001 | | |
| mFS 3-4 | | | 5 (3.0) | 51 (63.8) | < 0.001 | | |
| IVH | | | 24 (14.6) | 30 (37.5) | < 0.001 | | |
| BMI, kg/m^2^ | | | 23.6±2.8 | 24.1±2.8 | 0.156 | | |
| Glucose, mmol/L | | | 6.94±1.67 | 7.80±2.08 | 0.001 | | |
| SIH | | | 41 (25.0) | 33 (41.3) | 0.010 | | |
| TC, mmol/L | | | 4.97±1.08 | 4.55±0.94 | 0.006 | | |
| TG, mmol/L | | | 1.47±0.78 | 1.34±0.76 | 0.250 | | |
| HDL-C, mmol/L | | | 1.30±0.30 | 1.29±0.33 | 0.878 | | |
| LDL-C, mmol/L | | | 2.76±0.82 | 2.47±0.78 | 0.015 | | |
| Sodium, mmol/L | | | 138.9±3.5 | 139.4±3.7 | 0.268 | | |
| Potassium, mmol/L | | | 3.80±0.39 | 3.76±0.43 | 0.521 | | |
| Symptomatic vasospasm | | | 18 (11.0) | 42 (52.5) | < 0.001 | | |
| Delayed cerebral infarction | | | 8 (4.9) | 24 (30.0) | < 0.001 | | |
| Rebleeding | | | 0 (0) | 6 (7.5) | 0.001 | | |
| Hydrocephalus | | | 3 (1.8) | 15 (18.8) | < 0.001 | | |
| Seizure | | | 1 (0.6) | 2 (2.5) | 0.523 | | |
| Poor outcome at discharge | | | 124 (75.6) | 74 (92.5) | 0.002 | | |
| Poor outcome at 3 months | | | 12 (7.3) | 22 (27.5) | < 0.001 | | |
| Poor outcome at 12 months | | | 4 (2.4) | 15 (18.8) | < 0.001 | | |

**Table S1** Characteristics, complications, and outcomes of PMH and NPMH patients

SIH: stress-induced hyperglycemia; PMH: perimesencephalic subarachnoid hemorrhage; NPMH: non-perimesencephalic subarachnoid hemorrhage; HH: Hunt and Hess; mFS: modified Fisher scale; IVH: intraventricular hemorrhage; BMI: body mass index; TC: total cholesterol; TG: triglyceride; HDL-C: high-density lipoprotein cholesterol; LDL-C: low-density lipoprotein cholesterol
