# Supplementary material for: Effect of stress‐induced hyperglycemia after non‐traumatic non‐aneurysmal subarachnoid hemorrhage on clinical complications and functional outcomes
Source: CNS Neurosci Ther. 2022 Mar 15;28(6):942–52. doi: 10.1111/cns.13826 (PMC9062555; doi:10.1111/cns.13826)
Supplement: Supplementary file 5 — Table S2 [file CNS-28-942-s002.docx]

|  | **Symptomatic vasospasm** | |  | **Delayed cerebral infarction** | |  | **Hydrocephalus** | |
| --- | --- | --- | --- | --- | --- | --- | --- | --- |
| **Variable** | **OR (95% CI)** | **P value** |  | **OR (95% CI)** | **P value** |  | **OR (95% CI)** | **P value** |
| Age, yr | 1.026 (0.998-1.055) | 0.070 |  | 1.043 (1.004-1.083) | 0.029 |  | 1.032 (0.984-1.082) | 0.191 |
| Gender, female | 0.951 (0.529-1.712) | 0.868 |  | 1.304 (0.620-2.746) | 0.484 |  | 0.788 (0.295-2.105) | 0.634 |
| Alcohol | 0.673 (0.363-1.250) | 0.210 |  | 0.814 (0.373-1.776) | 0.605 |  | 0.784 (0.284-2.165) | 0.639 |
| Smoke | 0.698 (0.373-1.307) | 0.261 |  | 1.074 (0.498-2.316) | 0.856 |  | 0.663 (0.228-1.925) | 0.449 |
| Hypertension | 1.794 (0.984-3.269) | 0.056 |  | 4.126 (1.901-8.955) | < 0.001 |  | 4.551 (1.641-12.621) | 0.004 |
| NPMH | 8.965 (4.645-17.302) | < 0.001 |  | 8.357 (3.549-19.679) | < 0.001 |  | 12.385 (3.469-44.215) | < 0.001 |
| HH grade 3-5 | 12.737 (5.273-30.765) | < 0.001 |  | 17.349 (7.105-42.359) | < 0.001 |  | 9.762 (3.495-27.270) | < 0.001 |
| mFS 3-4 | 12.300 (6.142-24.632) | < 0.001 |  | 6.913 (3.139-15.225) | < 0.001 |  | 11.065 (3.744-32.700) | < 0.001 |
| IVH | 4.758 (2.478-9.134) | < 0.001 |  | 5.360 (2.458-11.689) | < 0.001 |  | 4.022 (1.510-10.716) | 0.005 |
| BMI, kg/m^2^ | 1.028 (0.925-1.141) | 0.611 |  | 0.946 (0.828-1.080) | 0.413 |  | 0.901 (0.759-1.069) | 0.231 |
| Glucose, mmol/L | 2.063 (1.618-2.630) | < 0.001 |  | 1.964 (1.558-2.476) | < 0.001 |  | 1.496 (1.232-1.816) | < 0.001 |
| SIH | 9.874 (5.095-19.135) | < 0.001 |  | 18.957 (6.920-51.933) | < 0.001 |  | 9.683 (3.067-30.574) | < 0.001 |
| TC, mmol/L | 0.935 (0.688-1.270) | 0.666 |  | 1.056 (0.707-1.576) | 0.792 |  | 1.203 (0.758-1.910) | 0.432 |
| TG, mmol/L | 1.100 (0.740-1.635) | 0.638 |  | 1.241 (0.758-2.033) | 0.390 |  | 0.819 (0.387-1.734) | 0.602 |
| HDL-C, mmol/L | 1.723 (0.630-4.709) | 0.289 |  | 0.773 (0.187-3.199) | 0.722 |  | 1.164 (0.227-5.983) | 0.856 |
| LDL-C, mmol/L | 0.855 (0.574-1.273) | 0.441 |  | 1.101 (0.657-1.846) | 0.715 |  | 1.253 (0.685-2.293) | 0.464 |
| Sodium, mmol/L | 1.041 (0.956-1.133) | 0.356 |  | 1.000 (0.901-1.111) | 0.994 |  | 0.955 (0.840-1.085) | 0.477 |
| Potassium, mmol/L | 0.543 (0.257-1.149) | 0.110 |  | 0.260 (0.096-0.702) | 0.008 |  | 0.486 (0.142-1.661) | 0.250 |

**Table S2** Univariate logistic regression analysis for predicting symptomatic vasospasm, delayed cerebral infarction, and hydrocephalus

NPMH: non-perimesencephalic subarachnoid hemorrhage; HH: Hunt and Hess; mFS: modified Fisher scale; IVH: intraventricular hemorrhage; BMI: body mass index; SIH: stress-induced hyperglycemia; TC: total cholesterol; TG: triglyceride; HDL-C: high-density lipoprotein cholesterol; LDL-C: low-density lipoprotein cholesterol; OR: odds ratio; Cl: confidence interval
