# Supplementary material for: Effect of stress‐induced hyperglycemia after non‐traumatic non‐aneurysmal subarachnoid hemorrhage on clinical complications and functional outcomes
Source: CNS Neurosci Ther. 2022 Mar 15;28(6):942–52. doi: 10.1111/cns.13826 (PMC9062555; doi:10.1111/cns.13826)
Supplement: Supplementary file 6 — Table S3 [file CNS-28-942-s005.docx]

|  | **Poor outcome at discharge** | |  | **Poor outcome at 3 months** | |  | **Poor outcome at 12 months** | |
| --- | --- | --- | --- | --- | --- | --- | --- | --- |
| **Variable** | **OR (95% CI)** | **P value** |  | **OR (95% CI)** | **P value** |  | **OR (95% CI)** | **P value** |
| Age, yr | 1.019 (0.991-1.048) | 0.188 |  | 1.019 (0.985-1.054) | 0.282 |  | 1.033 (0.986-1.082) | 0.172 |
| Gender, female | 0.752 (0.396-1.431) | 0.385 |  | 0.864 (0.414-1.802) | 0.696 |  | 0.310 (0.100-0.964) | 0.043 |
| Alcohol | 1.086 (0.559-2.107) | 0.808 |  | 0.625 (0.284-1.374) | 0.242 |  | 0.925 (0.351-2.441) | 0.875 |
| Smoke | 1.071 (0.547-2.099) | 0.841 |  | 1.114 (0.528-2.352) | 0.776 |  | 1.663 (0.649-4.263) | 0.289 |
| Hypertension | 0.916 (0.466-1.800) | 0.800 |  | 1.983 (0.952-4.132) | 0.068 |  | 2.410 (0.938-6.191) | 0.068 |
| NPMH | 3.978 (1.609-9.835) | 0.003 |  | 4.805 (2.234-10.332) | < 0.001 |  | 9.231 (2.952-28.862) | < 0.001 |
| HH grade 3-5 | 7.722 (1.024-58.233) | 0.047 |  | 10.263 (4.357-24.176) | < 0.001 |  | 11.389 (4.145-31.295) | < 0.001 |
| mFS 3-4 | 8.250 (1.933-35.211) | 0.004 |  | 4.385 (2.057-9.347) | < 0.001 |  | 7.052 (2.624-18.953) | < 0.001 |
| IVH | 7.836 (1.834-33.470) | 0.005 |  | 2.552 (1.180-5.518) | 0.017 |  | 3.600 (1.381-9.383) | 0.009 |
| BMI, kg/m^2^ | 1.053 (0.939-1.181) | 0.379 |  | 1.097 (0.961-1.252) | 0.171 |  | 1.022 (0.864-1.209) | 0.798 |
| Glucose, mmol/L | 1.255 (0.999-1.576) | 0.051 |  | 1.500 (1.257-1.789) | < 0.001 |  | 1.605 (1.314-1.961) | < 0.001 |
| SIH | 2.850 (1.210-6.712) | 0.017 |  | 3.570 (1.697-7.509) | 0.001 |  | 7.700 (2.660-22.292) | < 0.001 |
| TC, mmol/L | 1.013 (0.725-1.414) | 0.942 |  | 0.932 (0.635-1.368) | 0.720 |  | 0.971 (0.601-1.568) | 0.903 |
| TG, mmol/L | 0.913 (0.591-1.410) | 0.682 |  | 1.024 (0.619-1.694) | 0.925 |  | 0.966 (0.500-1.866) | 0.918 |
| HDL-C, mmol/L | 2.416 (0.707-8.257) | 0.159 |  | 2.066 (0.612-6.972) | 0.242 |  | 2.577 (0.580-11.448) | 0.213 |
| LDL-C, mmol/L | 0.969 (0.632-1.487) | 0.887 |  | 0.905 (0.553-1.480) | 0.690 |  | 0.904 (0.484-1.687) | 0.751 |
| Sodium, mmol/L | 1.015 (0.928-1.109) | 0.751 |  | 0.993 (0.897-1.098) | 0.885 |  | 0.973 (0.856-1.105) | 0.673 |
| Potassium, mmol/L | 1.400 (0.621-3.152) | 0.417 |  | 0.467 (0.183-1.189) | 0.110 |  | 0.605 (0.184-1.990) | 0.409 |

**Table S3** Univariate logistic regression analysis for predicting functional outcomes at discharge, 3 months, and 12 months

NPMH: non-perimesencephalic subarachnoid hemorrhage; HH: Hunt and Hess; mFS: modified Fisher scale; IVH: intraventricular hemorrhage; BMI: body mass index; SIH: stress-induced hyperglycemia; TC: total cholesterol; TG: triglyceride; HDL-C: high-density lipoprotein cholesterol; LDL-C: low-density lipoprotein cholesterol; OR: odds ratio; Cl: confidence interval
